# Sex Moderates the Mediating Effect of Physical Activity in the Relationship Between Dietary Habits and Sleep Quality in University Students

**DOI:** 10.3390/nu18010026

**Published:** 2025-12-20

**Authors:** Jarosław Domaradzki

**Affiliations:** Department of Biological Principles of Physical Activity, Wroclaw University of Health and Sport Sciences, 51-612 Wrocław, Poland; jaroslaw.domaradzki@awf.wroc.pl

**Keywords:** dietary behaviours, sleep quality, physical activity, mediation, sex differences, college students, indirect associations

## Abstract

**Background/Objectives**: Diet and physical activity are key lifestyle behaviours associated with sleep quality, yet their combined and sex-specific associations remain insufficiently understood. This study examined the associations between dietary behaviours and sleep quality among university students and assessed whether physical activity formed part of an indirect statistical association between these variables, with sex considered as a moderator. **Methods**: A cross-sectional study was conducted among 418 students (199 males, 219 females) from the Wroclaw University of Health and Sport Sciences. Body height and body mass were measured using standard anthropometric procedures. Sleep quality (SQ) was registered with the Pittsburgh Sleep Quality Index (PSQI), dietary habits were assessed with the Questionnaire of Eating Behaviours (QEB) and physical activity (PA) was assessed with the International Physical Activity Questionnaire (IPAQ). Data-driven feature-selection methods were applied to identify dietary behaviours associated with sleep quality, which were combined into a Synthetic Dietary Behaviour Index (SDBI). A moderated mediation model, adjusted for body mass index (BMI), was then used to examine the statistical associations between dietary behaviours, physical activity, sleep quality, and sex. Sleep quality was modelled as a continuous PSQI score in mediation analyses, while the dichotomised PSQI category was used only for feature selection. **Results**: Machine-learning feature selection identified nine dietary behaviours statistically associated with sleep quality. Unfavourable behaviours—fast food, fried meals, sweetened beverages, energy drinks and alcohol—were linked to poorer sleep, whereas vegetables, curd cheese and wholegrain bread were associated with better sleep. Poor sleep was more prevalent among females (45.2% vs. 14.6%, χ^2^ (1) = 65.4, *p* < 0.001). The mediation model indicated that physical activity formed part of a statistically significant but modest indirect association between dietary behaviour and sleep quality, with sex moderating the IPAQ → PSQI path (β = −0.45, *p* = 0.006). Indirect associations were significant for both sexes but stronger among females (males: β = 0.032, *p* = 0.021; females: β = 0.102, *p* = 0.004). **Conclusions**: Unfavourable dietary patterns and lower physical activity were statistically associated with poorer sleep quality, with a stronger indirect statistical effect observed among females. These findings support the relevance of integrated, sex-sensitive lifestyle approaches addressing both dietary behaviours and physical activity, while acknowledging the cross-sectional nature of the data.

## 1. Introduction

Sleep quality is strongly shaped by daily routines and lifestyle transitions common in university students, including altered schedules, increased cognitive demands, and changes in eating behaviours and physical activity [1,2,3,4].

Good health depends on many aspects of our daily lives, with sleep quality, eating habits, and physical activity being especially important. These lifestyle domains are closely interconnected and tend to co-occur within individuals. Young adults frequently display irregular eating patterns, fluctuating activity levels, and inconsistent sleep schedules, which may be jointly linked to adverse physical and psychological outcomes. Research consistently shows that diet, physical activity, and sleep each contribute to physical and mental well-being [5,6,7], yet their interactions remain complex. Sleep influences metabolic, hormonal, and emotional regulation [8,9]. Insufficient sleep has been linked to adverse outcomes, including weight gain, increased cardiovascular risk, and poorer mental health [10,11]. Among young adults, irregular sleep patterns frequently co-occur with unhealthy dietary behaviours and lower physical activity levels [12]. Importantly, recent studies from Central European populations further indicate that unfavourable sleep routines—particularly short sleep duration and late-night eating—are associated with higher BMI and less favourable somatic health indicators in young adults, underscoring the close interrelationship between sleep, nutrition, and cardiometabolic health [13].

What we eat also plays a big role in how well we sleep, through both body and mind. Diets that are packed with fruits, vegetables, whole grains, and healthy fats have been linked to better, longer sleep [14,15,16]. On the other hand, eating more refined carbs, saturated fats, and added sugars is tied to shorter, more restless nights [17,18]. Emerging evidence indicates that broader dietary patterns—including meal timing, variety, and regularity—may be related to sleep outcomes more effectively than individual nutrients [19].

Physical activity constitutes another key lifestyle component associated with sleep. Healthier dietary habits often co-occur with higher activity levels, and regular physical activity is linked with shorter sleep latency, better continuity, and improved restorative function [20,21,22]. Among university students, low activity and high sedentary time frequently coincide with stress and poor sleep. Given these co-occurring behaviours, physical activity may serve as a behavioural link connecting dietary choices with sleep quality, as healthier diets are typically accompanied by more regular activity [23].

Previous research has shown that sleep, diet, physical activity, and stress form interconnected behavioural clusters in young adults. Most prior studies have focused either on psychological mediators between physical activity and sleep or on direct diet–sleep or activity–sleep associations [24,25,26]. Very few have evaluated whether physical activity itself may function as an intermediate statistical pathway between dietary patterns and sleep quality [27]. Moreover, to our knowledge no study has examined this pathway while simultaneously assessing sex as a moderator, despite clear evidence of sex differences in lifestyle behaviours and sleep regulation. Together, the co-occurrence of dietary patterns, habitual physical activity, and sleep quality suggests that an integrated behavioural model may help clarify how these domains interact.

Beyond dyadic associations, growing evidence indicates that dietary behaviours, physical activity, sedentary time, and stress tend to co-occur and form clustered lifestyle patterns among university students [28]. A recent scoping review by Guerriero et al. (2025) synthesised findings in this population, demonstrating that low physical activity and high sedentary time are consistently associated with elevated stress and other unfavourable lifestyle behaviours, while also highlighting the lack of standardised, evidence-based intervention protocols [28,29]. This broader context reinforces the need to analyse diet, physical activity, and sleep within an integrated behavioural framework. Moreover, well-documented sex differences in metabolism, hormonal responses, and behavioural regulation suggest that these pathways may not operate identically across males and females [30,31].

Understanding these interrelations is especially important among college students, a population characterised by irregular eating schedules, poor sleep hygiene, and variable PA patterns [31]. Clarifying how these behaviours interact could inform targeted interventions for sleep and lifestyle health promotion.

This study integrates behavioural clustering, machine-learning-based feature selection, and moderated mediation analysis to elucidate the pathways linking diet, physical activity, and sleep quality in university students. Unlike previous studies focusing on isolated lifestyle behaviours, our approach examines their combined associations and tests whether physical activity forms part of an indirect statistical pathway linking diet and sleep. By incorporating sex as a moderator, the study captures inter-individual differences that may influence these behavioural pathways. This integrated framework provides a clearer understanding of how dietary patterns and physical activity jointly relate to sleep quality.

Therefore, the aim of the present study was to examine the associations between dietary behaviours and sleep quality among university students, with particular attention to the role of physical activity and sex differences. Specifically, the study aimed to (1) assess the association between sex and sleep quality; (2) identify dietary behaviours most strongly related to sleep quality; (3) examine whether physical activity formed part of an indirect statistical pathway linking dietary behaviour patterns and sleep quality; and (4) explore whether sex moderates the strength of this indirect pathway.

## 2. Materials and Methods

In this work, data from two independent student samples were combined into a single analytical dataset comprising only participants with complete information on dietary habits, physical activity, and sleep quality. Data were collected between 2022 and 2023 and included anthropometric assessments, body composition and balance measurements, as well as questionnaires related to injury occurrence, dietary habits, physical activity, sleep quality, mental health, quality of life, and socio-economic status. The final sample size differs from previous publications because only variables relevant to the present analysis were retained.

### 2.1. Study Design

This cross-sectional study was conducted among students of the Wroclaw University of Health and Sport Sciences in 2022–2023. Two independent student cohorts were collected during consecutive academic years, using identical recruitment procedures and measurement protocols. For the present analyses, the two cohorts were merged, and only participants with complete data on dietary behaviours, physical activity, and sleep quality were included.

All participants underwent anthropometric assessments and completed standardised questionnaires on dietary habits (QEB), physical activity (IPAQ), sleep quality (PSQI), and sociodemographic characteristics. The present study focuses specifically on the behavioural associations among diet, physical activity, and sleep quality. Although some variables from the broader dataset were used in unrelated publications, the current analysis is distinct, methodologically independent, and has not been published elsewhere.

### 2.2. Ethics

Ethical clearance was obtained from the Senate Research Ethics Committee of Wroclaw University of Health and Sport Sciences (reference number 13/2022). Before taking part in the research, each participant was fully informed about the study procedures and provided digital consent

### 2.3. Sample Size

The required sample size was estimated based on established recommendations for exploratory multivariate analyses. A conventional “rule of thumb” approach was applied, specifying that at least ten observations per predictor variable are necessary to ensure stable estimation of odds ratios in regression models [32,33,34]. Additionally, a complementary calculation was performed using a formula that incorporates the assumed margin of error (δ) [35]. The general expression for determining the sample size required to achieve a margin of error δ when estimating a true probability *p* at a 95% confidence level is as follows:

$$n=(\frac{1.96}{\delta }{)}^{2}\times p\left(1-p\right)$$

Assuming a small margin of error of 0.05 and the maximum variance condition (*p* = 0.5; worst-case scenario), the required sample size was estimated at 462 participants, accounting for an anticipated 20% dropout rate.

Although the initial target sample size was estimated at 462 participants, the final analytical sample comprised 418 students. This difference reflects the size of the available cohorts after applying predefined inclusion and exclusion criteria, as well as the requirement for complete data on key variables following the imputation procedure. The resulting sample size was considered sufficient for the main analyses, particularly given the heuristic nature of the “10 cases per predictor” rule and the use of continuous outcome measures in the primary models. Moreover, the observed sample size provided adequate precision for estimating the main regression and moderated mediation models, and no indications of model instability were observed.

To mitigate potential small-sample bias, a sensitivity analysis using Firth’s bias-reduced logistic regression was additionally conducted [36].

### 2.4. Participants

A total of 454 students were initially recruited across both cohorts. Of these, 36 students were removed because of missing PSQI, SES, or balance measures, and 13 had incomplete data restricted to a single questionnaire or balance measurement; these case were handled using the imputation procedure described in Section 2.8. The final analytical sample consisted of 418 participants (199 men, 219 women) with complete data on dietary behaviours, physical activity, and sleep quality.

**Figure 1:**
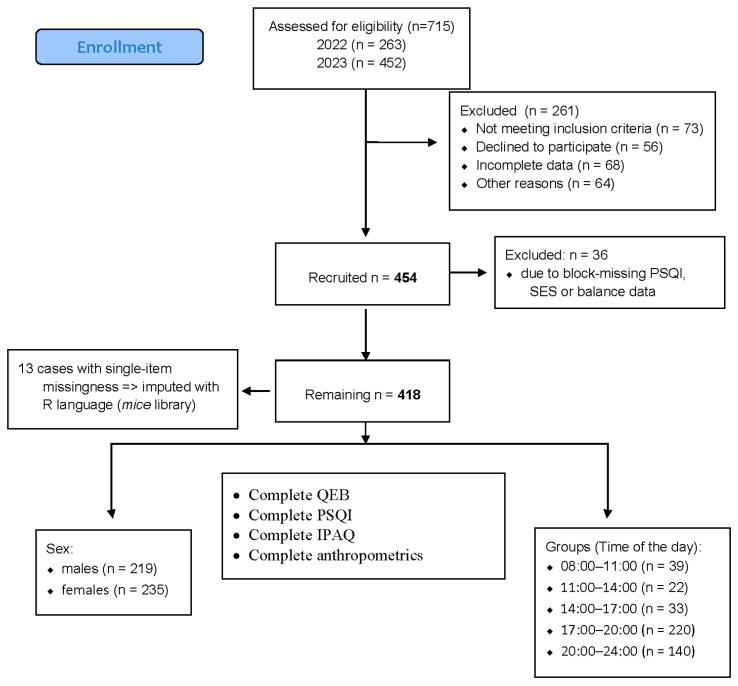
Flow diagram of the progress through the all phases of data collection.

Figure 1 presents an updated flow diagram specific to the current study, ensuring that all numbers correspond directly to the analytical sample used in the results.

Preliminary inclusion criteria required participants to be physically active students regularly attending in-person university classes. Exclusion criteria encompassed individuals participating in university-regulated competitive sports or enrolled in specialised sport or elite performance programmes. Inclusion criteria were: (1) age 18–25 years, (2) full-time university enrolment, (3) participation in regular in-person academic activities, and (4) the absence of chronic conditions known as related to sleep, diet, or physical activity. Exclusion criteria included: diagnosed sleep disorders, chronic metabolic or psychiatric conditions, night-shift work, and implausible questionnaire responses (e.g., extreme dietary frequencies or improbable MET-min/week values). These criteria were applied prior to imputation.

The resulting analytical sample of 418 participants provided complete anthropometric, dietary, physical activity, and sleep data.

Although the current study uses data originating from the same institutional cohorts as earlier publications, earlier work addressed unrelated research questions (e.g., injury risk, motor coordination) and used different variables. The present analysis is methodologically independent and has not been published elsewhere. The present analyses are therefore novel and analytically independent.

To confirm that the two cohorts could be combined, we compared them on age, sex distribution, BMI, PSQI, QEB dietary items and IPAQ scores. Independent t-tests and chi-square tests showed no significant differences between cohorts (all *p* > 0.05), supporting their equivalence and justifying the merged analytical sample.

### 2.5. Anthropometric Measurements

Anthropometric measurements were carried out in the Biokinetics Research Laboratory, part of the Central Research Laboratory at the Wroclaw University of Health and Sport Sciences.

Body height was measured twice to the nearest 0.1 cm using a GPM anthropometer (GPM instruments GmBH, Susten, Switzerland). Body mass was measured to the nearest 0.1 kg using an InBody230 device (InBody Co., Ltd., Cerritos, CA, USA), which was used solely as an electronic scale. No bioelectrical impedance or body composition parameters were analysed in the present study. Body Mass Index (BMI) was used only for descriptive characterisation of the study sample, and has been calculated from these measurements:

$$BMI=\frac{\mathrm{body}\;\mathrm{mass}\;\left[\mathrm{kg}\right]}{\mathrm{body}\;\mathrm{height}{\;[m}^{2}]}$$

### 2.6. Questionnaire Measurements

#### 2.6.1. Sleep Quality—Pittsburgh Sleep Quality Index (PSQI)

Sleep quality was evaluated using the Pittsburgh Sleep Quality Index (PSQI) [37] a widely validated self-report questionnaire assessing sleep patterns over the previous month. The questionnaire includes 19 items yielding a global score from 0 to 21, with higher values indicating poorer sleep quality.

A total score ≤5 indicated good sleep quality, and >5 indicated poor sleep quality, consistent with established cut-offs.

#### 2.6.2. Physical Activity—International Physical Activity Questionnaire (IPAQ)

Physical activity was assessed using the Polish adaptation of the International Physical Activity Questionnaire—Long Form (IPAQ-LF) [38], administered electronically via Google Forms. Responses were converted into metabolic equivalent minutes per week (MET-min/week) to estimate total and domain-specific physical activity, as well as sedentary time. In the present study, physical activity data were used as a behavioural variable within the analyses rather than for training-load classification.

#### 2.6.3. Dietary Intake Questionnaire—Questionnaire Eating Behaviours (QEB)

Dietary intake over the past 12 months was assessed using the self-administered Questionnaire of Eating Behaviours (QEB) [39], a validated food frequency tool (Fleiss’ kappa = 0.64–0.84). From the original 21 items, the recommended 16-item core set was applied [40]. Consumption was recorded on a six-point scale (“never” to “several times/day”) and converted to average daily intake values. For the present study, all 16 core items were included as candidate dietary variables for the subsequent feature-selection procedures. In this study, QEB items were used solely as dietary-behaviour indicators within the diet–sleep analytical framework.

### 2.7. Handling and Imputation of Missing Data

Missing data occurred in three variables: four students had incomplete QEB responses, six had missing PSQI values, and three had missing balance measurements. A logistic-regression Missing Completely At Random (MCAR) test indicated that missingness did not depend on observed variables, supporting the assumption that data were MCAR [41,42]. Balance variables were not included in diagnostics because they were not used in subsequent analyses.

Given the requirement for complete datasets in multivariable procedures, missing values were imputed using multiple imputation by chained equations (MICE) implemented in R (mice v3.14.0). Twenty imputations (m = 20) were generated using an imputation model that included all variables used in subsequent analyses. Multiple imputation was performed prior to analyses. Moderated mediation models were estimated separately within each imputed dataset using regression-based procedures equivalent to PROCESS Model 14. Indirect effects were obtained within each imputation using non-parametric bootstrapping, and parameter estimates were subsequently pooled across imputations according to Rubin’s rules. This approach ensures that both point estimates and confidence intervals appropriately reflect uncertainty arising from missing data. All imputation procedures were conducted using the mice package in R, while moderated mediation analyses were implemented using standard regression modelling functions applied iteratively across imputed datasets. Pooling was applied to path coefficients and indirect effects; bootstrap confidence intervals were derived within each imputed dataset prior to pooling. Predictive mean matching was applied for continuous variables, and appropriate regression models were used for categorical items. Convergence diagnostics confirmed stable imputation chains.

### 2.8. Statistics

The statistical analysis followed a three-stage analytical framework.

Stage 1: Feature selection.

All 16 QEB dietary variables were screened using two complementary procedures: the Boruta algorithm and LASSO regression. Both were applied to standardised variables with sleep-quality category as the reference outcome to identify dietary items most strongly associated with sleep quality. Boruta was used as an initial, conservative screening procedure to identify dietary behaviours showing robust associations with sleep quality across random forest iterations, thereby reducing the likelihood of spurious selections. LASSO regression was subsequently applied as a parsimonious discrimination step to retain the most informative dietary variables while accounting for multicollinearity and limiting model complexity. The combination of both methods was intended to balance robustness and interpretability in feature selection.

Feature selection and discrimination were conducted on the full analytical dataset, without an independent train–test split.

Stage 2: Construction of the Synthetic Dietary Behaviour Index (SDBI).

Dietary variables consistently selected by both feature-selection methods were aggregated into a composite index using the non-pattern variant of Multidimensional Comparative Analysis (MCA). Higher values indicated more unfavourable dietary profiles. Robustness was evaluated using Principal Component Analysis (PCA) and Rceiver-Operating-Characteristic (ROC) analysis applied to the same variables. These procedures were intended to assess internal consistency and discriminative capacity of the index rather than predictive performance in an independent sample.

Stage 3: Moderated mediation analysis.

Rather than relying on the stepwise causal-testing framework originally proposed by Baron and Kenny [43], the analysis applied contemporary regression-based moderated mediation procedures implemented in PROCESS Model 14 [44,45].

The SDBI served as the independent variable (X), physical activity (IPAQ total MET-min/week) as the mediator (M), and sleep quality (PSQI score) as the dependent variable (Y). Sex was included as a moderator on the M → Y path, and BMI was entered as a covariate.

Indirect and conditional indirect associations were estimated using bias-corrected bootstrapping with 5000 resamples. Effects were interpreted as statistical associations given the cross-sectional design. Model diagnostics for the linear regression components of the moderated mediation analysis were evaluated using standard procedures, including inspection of residual distributions and assessment of homoscedasticity. No substantial deviations from model assumptions were observed, and therefore no additional corrective measures were required.

Exploratory analyses.

All variables were transformed using the Yeo–Johnson transformation [46] and standardised prior to all cluster analyses. Missing data were handled using multiple imputation (m = 20), and pooled parameter estimates were computed using Rubin’s rules. Hierarchical clustering and tanglegram comparisons of dietary patterns between sleep-quality groups were conducted for descriptive purposes and are presented in the Supplementary Materials, using the dendextend framework [47,48].

Two-way ANOVA and independent-sample *t*-tests were used only for descriptive comparisons. All analyses were conducted in R (v4.3) and Statistica 13.5, with *p* < 0.05 considered statistically significant.

## 3. Results

### 3.1. General Characteristics

Descriptive statistics for demographic, anthropometric and questionnaire variables are presented in Supplementary Table S1. Due to skewed distributions for several dietary variables (QEB items), the Yeo–Johnson transformation was applied to all food-frequency scores. Age, BMI, PSQI and IPAQ scores were analysed in their original scale.

The two cohorts did not differ significantly in age, sex distribution, BMI, PSQI total score, IPAQ MET-min/week or any of the 16 QEB items (all *p* > 0.10), supporting their combination into a single analytical sample.

Table 1 summarises the characteristics of the final sample by sex. Men had higher body height, body mass and BMI than women (all *p* < 0.001) and reported higher total physical activity (IPAQ MET-min/week, *p* < 0.001). Women showed significantly higher PSQI scores (indicating poorer sleep quality; *p* < 0.001).

Clear sex differences were also observed in dietary behaviours. Compared with men, women more frequently consumed wholegrain bread, milk, fermented milk products, curd cheese, legumes, fruits and vegetables (all *p* ≤ 0.02). Men more often reported consumption of fast food, fried meals, yellow cheese, canned meals, sweetened beverages, energy drinks and alcoholic drinks (all *p* < 0.001). No significant sex differences were found for fish or sweets (*p* > 0.05).

Overall, these findings indicate distinct sex-related profiles of diet, physical activity and sleep quality, supporting the decision to treat sex as an important stratification and moderation factor in subsequent analyses.

### 3.2. Congruence and Divergence in Dietary Patterns by Sleep Quality

Sex was significantly associated with sleep-quality category (good vs. poor sleepers; χ^2^ (1) = 65.42, *p* < 0.001, φ = 0.40). Poor sleep was reported by 14.6% of men and 45.2% of women, indicating a substantially higher prevalence of poor sleep quality among female students.

**Figure 2:**
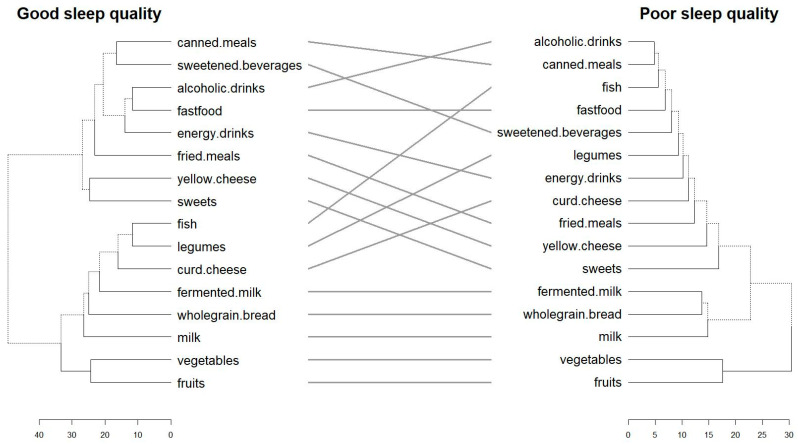
Two-face tanglegram comparing hierarchical clustering of dietary behaviours between participants with good (left) and poor (right) sleep quality. Each tree represents the structure of sex-adjusted residualized dietary items after Yeo–Johnson transformation and standardisation. Lines connecting the trees indicate corresponding dietary variables. Parallel (horizontal) connections reflect structural congruence, while crossed lines highlight divergences in the relative positioning of dietary clusters between groups. Scale bars below dendrograms presented distances between items and clusters.

Figure 2 presents a tanglegram illustrating similarities and differences in dietary pattern clustering between good and poor sleepers. In both groups, healthier dietary items tended to cluster together, while less favourable food choices formed a separate cluster. However, the dietary pattern among poor sleepers appeared more fragmented, with weaker and less coherent grouping of food items compared with good sleepers. This visualisation suggests that, beyond individual dietary behaviours, overall dietary organisation differs between sleep-quality groups.

In both sleep-quality groups, vegetables, fruits, milk, fermented milk, and wholegrain bread formed a coherent “healthy” cluster, whereas fast food, fried meals, and yellow cheese clustered together as higher-fat or more processed foods. However, several local differences were evident. Among good sleepers, sweetened beverages, alcoholic drinks, and energy drinks tended to cluster more closely with other high-calorie items, whereas in poor sleepers these items appeared more dispersed. Fermented milk and curd cheese were more consistently grouped with plant-based foods among good sleepers than among poor sleepers. Quantitative indices describing similarity and divergence between clustering structures are reported in the Supplementary Materials (Table S3).

Taken together, these results suggest that although overall “healthy” and “unhealthy” dietary patterns are observed in both groups, poor sleepers exhibit a less coherent and more fragmented organisation of dietary habits. This provided the rationale for using feature-selection methods to identify specific dietary behaviours most strongly distinguishing good and poor sleepers.

### 3.3. Key Dietary Behaviours Distinguishing Good and Poor Sleepers

Building on the clustering results, machine-learning feature-selection procedures were applied to identify the dietary items most strongly associated with sleep-quality category.

**Figure 3:**
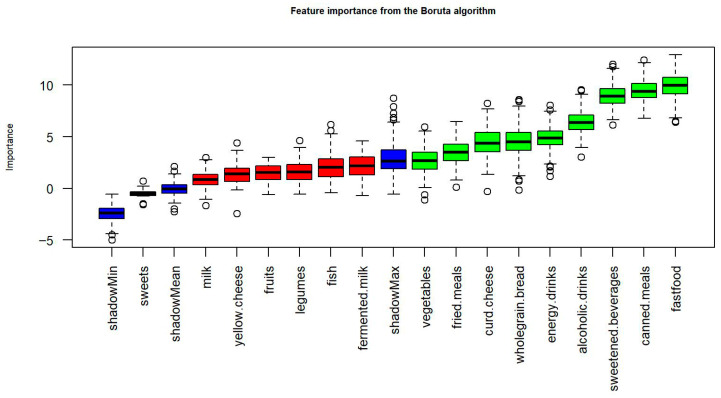
Feature importance of dietary behaviour variables identified by the Boruta algorithm. Green boxplots indicate variables confirmed as significant variable associated with sleep quality, red ones denote rejected variables, and blue boxplots represent shadow attributes generated for reference. The plot shows the distribution of variable importance scores across 500 random forest iterations.

The Boruta algorithm confirmed nine QEB items as important in distinguishing good vs. poor sleep (Figure 3, Table 2). Unfavourable behaviours included higher consumption of fast food, fried meals, canned meals, sweetened beverages, energy drinks and alcoholic drinks. Favourable behaviours included more frequent consumption of vegetables, curd cheese and wholegrain bread. The remaining seven items (milk, fermented milk, fish, legumes, fruits, yellow cheese and sweets) were rejected as non-informative in this classification context.

**Figure 4:**
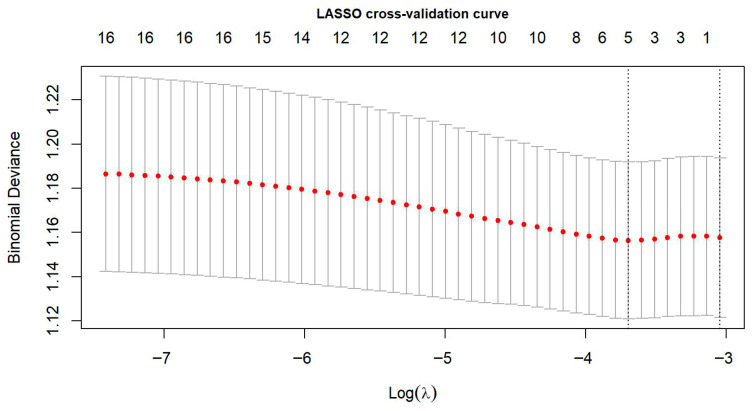
LASSO cross-validation curve for logistic regression predicting sleep quality. The red dots represent the mean binomial deviance (±1 SE) obtained across 10 cross-validation folds for different values of the regularisation parameter λ. The λ_1_se value was selected for the final model to ensure optimal parsimony and generalizability. Footnote: Red dots represent mean binomial deviance across 10 folds; vertical grey bars indicate ±1 standard error. The two vertical dashed lines indicate λ_min (left), corresponding to the minimum deviance, and λ_1_se (right), representing the most regularised model within one standard error of the minimum.

LASSO logistic regression with 10-fold cross-validation (Figure 4, Table 3) yielded a more parsimonious set of six dietary variables. Using the λ_1_se criterion, sweetened beverages, energy drinks, fast food and fried meals were retained as positively associated with poor sleep quality, whereas vegetables and curd cheese were negatively associated with poor sleep. Corresponding odds ratios indicated that higher intake of high-calorie or stimulant-containing foods was related to greater odds of poor sleep, while higher intake of vegetables and curd cheese was related to lower odds of poor sleep.

Across both selection procedures, six dietary items consistently emerged as key discriminative features: fast food, fried meals, sweetened beverages, energy drinks, vegetables and curd cheese. These items were therefore used to construct the Synthetic Dietary Behaviour Index (SDBI) for subsequent analyses.

In sensitivity analyses using Firth’s bias-reduced logistic regression, effect estimates were conservative and confidence intervals were wide; only curd cheese retained a statistically significant protective association, while all other predictors showed attenuated or non-significant effects (Supplementary Table S2).

### 3.4. Construction and Validation of a Multidimensional Synthetic Dietary Behaviour Index (SDBI)

For interpretative clarity, higher SDBI values indicate a more unfavourable dietary behaviour profile with respect to sleep quality, characterised by more frequent consumption of fast food, fried meals, sweetened beverages, and energy drinks, alongside lower intake of vegetables and curd cheese. Conversely, lower SDBI values reflect a dietary pattern more closely aligned with behaviours associated with better sleep quality. The SDBI was constructed using the six dietary variables consistently identified by Boruta and LASSO (fast food, fried meals, sweetened beverages, energy drinks, vegetables and curd cheese). In line with their observed associations with poor sleep, fast food, fried meals, sweetened beverages and energy drinks were coded as unfavourable dietary behaviours, whereas vegetables and curd cheese were coded as favourable, protective behaviours. The index was derived using the non-pattern variant of multidimensional comparative analysis (MCA), with higher SDBI scores reflecting a more unfavourable dietary profile with respect to sleep quality.

An alternative index based on principal component analysis (PCA) was also computed using the same set of variables. The correlation between the MCA- and PCA-based indices was low to moderate (Pearson r = 0.26, *p* < 0.001; Spearman ρ = −0.02, *p* = 0.69), suggesting that the two approaches captured partly different aspects of dietary behaviour.

Both indices differed significantly between good and poor sleepers, but with different magnitudes of separation. The MCA-derived SDBI showed a small yet significant difference between sleep-quality groups (t = −2.11, *p* = 0.036, η^2^ = 0.009). ROC analysis indicated modest but above-chance discrimination (AUC = 0.57, 95% CI 0.51–0.63). In contrast, the PCA-based index showed poorer discriminative performance (AUC = 0.37, 95% CI 0.31–0.42), with the DeLong test confirming that the difference between AUCs was statistically significant (*p* < 0.001). Taken together, these results indicate that although the SDBI performed better than the PCA-based index, its discriminative ability and between-group separation were modest in magnitude.

On this basis, the MCA-derived SDBI was selected as the primary dietary variable for the moderated mediation analyses, as it provided more interpretable and statistically useful—albeit modest—differentiation between good and poor sleepers.

### 3.5. Moderated Mediation Analysis: The Interplay Between Dietary Behaviours, Physical Activity, and Sleep Quality

The moderated mediation model (PROCESS Model 14) examined statistical pathways linking dietary behaviour (SDBI), physical activity (IPAQ total MET-min/week), sleep quality (PSQI total score), sex and BMI (Table 4).

#### 3.5.1. Path-Specific Regression Models

a-path: SDBI → physical activity

Higher SDBI scores (more unfavourable dietary patterns) were associated with lower physical activity levels (β = −0.16, SE = 0.05, t = −3.27, *p* = 0.001, 95% CI [−0.26, −0.06]). BMI was also negatively related to IPAQ (β = −0.22, *p* < 0.001), and sex showed a strong association (β = −0.67, *p* < 0.001), with men reporting higher activity levels. The model explained 14% of the variance in physical activity (R^2^ = 0.14).

b-path: physical activity → sleep quality (moderated by sex)

Higher physical activity was associated with better sleep quality (lower PSQI scores; β = −0.20, SE = 0.10, t = −2.05, *p* = 0.041, 95% CI [−0.40, −0.004]). The IPAQ × sex interaction was also significant (β = −0.45, SE = 0.16, t = −2.78, *p* = 0.006, 95% CI [−0.76, −0.14]), indicating that the association between physical activity and sleep quality was stronger among women. BMI (β = 0.91, *p* < 0.001) and sex (β = 2.42, *p* < 0.001) remained significant covariates. This model accounted for 44% of the variance in PSQI (R^2^ = 0.44).

c′-path: direct association between SDBI and sleep quality

The direct association between SDBI and PSQI, after accounting for physical activity, BMI and sex, was not statistically significant (β = −0.11, SE = 0.08, t = −1.35, *p* = 0.177, 95% CI [−0.27, 0.05]).

#### 3.5.2. Conditional Indirect Associations

Bootstrapped conditional indirect associations (Table 5) showed that the pathway from SDBI to PSQI via physical activity was statistically significant for both sexes. Among men, the indirect association was β = 0.032 (95% CI [0.003, 0.079], *p* = 0.021), and among women β = 0.102 (95% CI [0.023, 0.193], *p* = 0.004). In both cases the confidence intervals did not include zero, indicating a significant indirect statistical pathway linking more unfavourable dietary patterns with poorer sleep quality through lower physical activity. The larger indirect association among women suggests that this pathway is more pronounced in female students.

Overall, these results indicate that students with more unfavourable dietary profiles tend to report lower physical activity levels, which in turn are associated with poorer sleep quality. The indirect association between diet and sleep via physical activity is present in both sexes but appears stronger in women, while BMI remains an independent correlate of poorer sleep.

## 4. Discussion

The aim of this study was to examine the associations between dietary behaviours and sleep quality among university students and to evaluate whether physical activity formed part of an indirect statistical pathway linking these variables, as well as whether this pathway differed by sex. The analyses confirmed significant sex differences in sleep quality, with poorer sleep more prevalent among females. Machine-learning procedures (Boruta and LASSO) identified a consistent set of dietary behaviours that differentiated good and poor sleepers. Physical activity was part of a statistically significant indirect association between diet and sleep, and this indirect pathway was moderated by sex. Together, these findings support a multidimensional behavioural model in which unfavourable dietary habits are associated with lower physical activity and, in turn, poorer sleep quality, particularly among female students.

### 4.1. Sex Differences in Sleep Quality

Females demonstrated substantially poorer sleep quality than males, consistent with previous reports in student and young adult populations [14,49]. Our findings extend this evidence by showing that sex differences are also apparent within the behavioural pathways linking diet, physical activity and sleep quality. Specifically, women not only reported higher PSQI scores but also showed a stronger indirect statistical association between dietary behaviour and sleep quality through physical activity (Table 5). This pattern suggests that lifestyle-related behaviours may be associated differently to sleep outcomes in males and females.

These observations are in line with prior work indicating that sex differences in sleep may reflect a combination of biological, hormonal and psychosocial factors [28,50,51]. The present results add a behavioural component to this picture, indicating that health-related behaviours such as diet and physical activity may form part of sex-specific profiles of sleep vulnerability in late adolescence and early adulthood.

### 4.2. Dietary Patterns and Sleep Quality

The present findings indicate that specific components of dietary behaviour—rather than diet as a global construct—are strongly associated with sleep quality. By integrating cluster-based diagnostics with feature-selection methods, we showed that both the magnitude and the organisation of dietary habits differ between good and poor sleepers.

Machine-learning analyses consistently highlighted a small set of behaviours that differentiated sleep-quality groups. Poor sleepers more frequently consumed fast food, fried meals, sweetened beverages, energy drinks and alcohol, whereas good sleepers reported higher intake of vegetables, curd cheese and wholegrain bread. The six items retained by both Boruta and LASSO (fast food, fried meals, sweetened beverages, energy drinks, vegetables and curd cheese) formed the basis of the SDBI and reflect a coherent pattern in which high-calorie and stimulant-containing products are contrasted with plant-based and protein-rich foods. This pattern is compatible with earlier findings linking diets rich in saturated fat, refined carbohydrates and stimulants with shorter or more fragmented sleep [52,53].

Overall, the discriminative strength of this dietary pattern should be interpreted with caution. Although the SDBI differed significantly between good and poor sleepers and outperformed the PCA-based index, its discriminative performance and between-group effect size were modest, underscoring that dietary behaviours represent only one component of a broader behavioural context underlying sleep quality. Moreover, the dietary indicators used in this study capture frequency of consumption rather than detailed nutrient composition or timing. As a result, mechanistic interpretations—such as the roles of macronutrient balance, glycaemic load, or caffeine content—remain indirect and should be viewed as hypothesis-generating rather than confirmatory [52,53,54,55,56]. Frequency-based measures are well suited for behavioural profiling and screening; however, future studies combining such tools with nutrient-level data or objective dietary assessment (e.g., food diaries, biomarkers) may help clarify which specific nutritional pathways are most relevant for sleep outcomes in young adults.

### 4.3. Physical Activity as a Mediator Between Diet and Sleep

Previous research has shown that dietary and physical activity patterns often co-occur, forming lifestyle clusters that tend to track within individuals over time [57]. Our results are consistent with this perspective: students with healthier dietary profiles reported higher physical activity levels, and higher activity levels were associated with better sleep quality. When modelled jointly, physical activity formed part of an indirect statistical pathway linking dietary behaviour and sleep quality, and this pathway was present in both sexes.

The current literature on university students increasingly emphasises the joint role of physical activity, sedentary behaviour and stress in shaping sleep and mental health, but relatively few studies have formally examined physical activity as an intermediate statistical pathway in the diet–sleep relationship, particularly with sex-specific moderation. Most prior work has focused either on physical activity moderating the association between sleep and mental health outcomes [58,59] or on psychological variables mediating links between sleep and physical fitness [24]. The present study therefore adds to this literature by positioning physical activity as a behavioural bridge between diet and sleep quality in a sex-sensitive framework, rather than as an isolated correlate.

The mediation analysis indicated that students with healthier dietary behaviours tended to be more physically active, and that higher activity levels were statistically associated with better sleep outcomes. These findings are compatible with previous reports showing that regular physical activity is associated with improved sleep quality, reduced sleep latency and more favourable sleep architecture [26,60,61,62]. Within this behavioural context, physical activity may be viewed as part of a broader lifestyle profile that links diet and sleep, rather than as a single, isolated determinant. It should also be noted that the proportion of variance explained in physical activity was relatively modest (R^2^ = 0.14), indicating that a substantial share of variability in physical activity remains unexplained by dietary behaviours, BMI, and sex. This finding reinforces the interpretation of physical activity as one component within a broader and multifactorial lifestyle context influencing sleep quality. This observation is consistent with the notion that physical activity is influenced by a wide range of social, environmental, and psychological factors beyond diet.

The present results also fit within the broader behavioural literature on university students, in which physical activity, sedentary behaviour, and stress frequently cluster together. A recent scoping review by Guerriero et al. (2025) reported that low physical activity and high sedentary time are consistently associated with elevated stress and less favourable psychological outcomes in this population [28]. This behavioural clustering provides a plausible context for understanding why physical activity may form part of the statistical pathway linking dietary behaviours with sleep quality, rather than acting independently. In our study, this indirect pathway was more pronounced among females, suggesting that physical activity may play a relatively stronger behavioural role in linking dietary habits with sleep quality in women. Physical activity may support sleep not only through physiological processes—such as improved recovery or circadian alignment—but also by interacting with stress-related behavioural patterns that are commonly observed in university students.

### 4.4. Moderating Effect of Sex in the Mediation Pathway

To our knowledge, no previous studies have simultaneously examined physical activity as an intermediate pathway between dietary behaviours and sleep quality while also modelling the moderating role of sex. Most prior work has addressed these lifestyle domains separately—for example, by testing the moderating effect of physical activity on associations between sleep and mental health [62,63] or by examining psychological well-being as a mediator between sleep and physical fitness [23]. The present study therefore extends this literature by demonstrating that sex conditions the strength of the indirect pathway linking diet, physical activity and sleep.

In our analysis, the indirect association between dietary behaviour and sleep quality through physical activity was statistically significant in both sexes, but larger among females (Table 5). This suggests that physical activity may play a relatively stronger behavioural role for women within this diet–sleep pathway. Several mechanisms may be related to this pattern, including sex-related differences in stress regulation, circadian sensitivity and hormonal fluctuations [25,64,65], although such interpretations remain speculative and the present findings reflect statistical associations rather than causal processes.

Overall, these results indicate that the behavioural link between diet, physical activity and sleep quality may differ in intensity rather than direction across sexes. Future studies should investigate whether additional factors—such as chronotype, perceived stress or menstrual-cycle timing—further modify these sex-specific behavioural pathways.

### 4.5. Integrative Perspective and Behavioural Implications

Taken together, the findings support an integrative behavioural model in which dietary habits, physical activity and sleep quality are interrelated components of a broader lifestyle system. This triadic perspective highlights that sleep health should not be considered in isolation but rather in conjunction with daily behaviours related to nutrition and movement [6,23]. From a public-health standpoint, interventions designed to improve sleep in university students may be more effective when they simultaneously promote healthier eating and regular physical activity, rather than addressing each behaviour separately.

The present results also align with the wider lifestyle–mental-health framework described by Guerriero et al. (2025) [28], who emphasised the central role of physical activity as a feasible strategy for stress management and mental well-being in university students, while sedentary patterns were consistently associated with less favourable psychological outcomes. Recent scoping evidence highlights that these behaviours tend to co-occur rather than act in isolation, reinforcing the relevance of integrated lifestyle approaches in this population. As diet, physical activity, sleep and stress frequently co-occur, the current findings suggest that unfavourable dietary patterns and lower activity levels may form part of a broader behavioural profile associated with poorer sleep and potentially elevated stress. These observations reinforce the value of integrated behavioural approaches that jointly target movement, nutrition, sleep hygiene and stress regulation.

In practical terms, enhancing sleep quality among students with unhealthy dietary patterns may benefit from interventions that include accessible physical-activity opportunities alongside dietary guidance and stress-management strategies. Our moderated mediation results further indicate that such interventions may require sex-sensitive tailoring. Because the indirect pathway linking diet to sleep via physical activity was stronger among female students, addressing sex-specific activity barriers or incorporating components focused on emotional regulation may enhance programme effectiveness. Tailored, integrated strategies may therefore offer a more holistic and impactful approach to student health promotion.

Highlights. This study advances current understanding of the links between diet, physical activity and sleep quality in several ways. First, it integrates hierarchical clustering, machine-learning–based feature selection (Boruta and LASSO) and multidimensional index construction to characterise dietary behaviour patterns using a comprehensive, data-driven approach rather than relying on single food items or nutrients. Second, it applies a moderated mediation framework to examine how dietary patterns relate to sleep quality through physical activity, and whether this indirect association differs by sex, while adjusting for BMI. Third, by validating a synthetic dietary behaviour index (SDBI) derived from two independent methods and evaluating its discriminative performance, the study provides a methodological template for future multidimensional lifestyle analyses. Collectively, these elements offer a systems-oriented framework that links diet, physical activity and sleep quality in young adults.

Limitations. Several limitations should be considered when interpreting these findings. First, the cross-sectional design does not allow determination of temporal or causal direction in the diet–physical activity–sleep pathways. Moderated mediation analyses identify statistical associations but cannot establish causal sequencing.

Second, all behavioural measures—dietary intake (QEB), physical activity (IPAQ), and sleep quality (PSQI)—were assessed using self-report questionnaires, which are susceptible to recall bias, social desirability, and misclassification of frequency or intensity. In addition, previous research suggests that reporting accuracy may differ by sex, with men more likely to overestimate physical activity and women potentially reporting dietary behaviours and sleep quality with greater sensitivity. Such differences could have influenced the observed sex-specific associations and should be considered when interpreting the results. The absence of objective indicators (e.g., accelerometery, actigraphy, nutrient biomarkers) limits the precision with which behavioural or physiological mechanisms can be described.

Third, the study was conducted within a single specialised university of health and sport sciences. Students in this setting may differ from the broader population in baseline physical activity, body composition and health literacy, which may limit generalisability to more diverse groups. Moreover, the sample was restricted to physically active students regularly attending in-person classes, which may further limit the external validity of the findings. The observed associations may not generalise to more sedentary students or to individuals enrolled in non-health-related academic programmes, whose lifestyle patterns and sleep behaviours may differ substantially.

Fourth, the analytical framework was methodologically complex, combining feature selection, multidimensional index construction and moderated mediation. Although the use of Boruta, LASSO and multiple imputation reduces the risk of overfitting and bias due to missing data, the absence of external validation in an independent or longitudinal cohort remains a constraint. Replication in larger or multicentre datasets would strengthen confidence in the robustness of these pathways. Because feature selection and discrimination were performed on the same dataset, some degree of overfitting cannot be fully excluded; however, this risk was mitigated through the use of the conservative Boruta procedure, LASSO regularisation, and additional sensitivity analyses assessing the robustness of the selected dietary behaviours.

Finally, dietary assessment focused on frequency of intake rather than detailed nutrient composition or timing. As a result, mechanistic interpretations should be regarded as indirect and exploratory rather than confirmatory. Future studies incorporating objective dietary and sleep measures would help clarify the physiological processes underlying these behavioural associations.

## 5. Conclusions

The novelty of this study lies in its integrative, data-driven framework combining machine learning, multidimensional index construction and moderated mediation modelling to describe how dietary and physical activity behaviours jointly relate to sleep quality in young adults. Poor sleep quality was more common among females, and both dietary patterns and physical activity emerged as key behavioural correlates of sleep. Physical activity formed part of a statistically significant indirect association between dietary behaviour and sleep quality, and this indirect pathway was stronger among females.

Machine-learning feature selection identified a distinct set of dietary behaviours associated with poor sleep quality, characterised by higher intake of high-calorie and stimulant-containing foods and lower intake of nutrient-rich items. These findings highlight specific, modifiable dietary targets that may be relevant for sleep improvement in university populations.

Overall, the results suggest that supporting healthier dietary habits and increasing physical activity may help mitigate unfavourable sleep outcomes, particularly among females. These findings underscore the value of integrated, sex-sensitive approaches that simultaneously address nutrition and movement when designing lifestyle-based strategies to improve sleep quality in young adults.

## Figures and Tables

**Table 1:** Descriptive statistics of anthropometric characteristics and questionnaire scores (PSQI, QEB, IPAQ) by sex. Data for QEB have been transformed with Yeo-Johnson power transformation.

	Sex = Males, N = 199				Sex = Females, N = 219					
Variables	Mean	95%CI		SD	Mean	95%CI		SD	t	*p*
		Lower	Upper			Lower	Upper			
Body height [cm]	182.19	181.20	183.18	7.10	168.17	167.37	168.97	6.01	**21.85**	**0.000**
Body weight [kg]	79.63	78.25	81.00	9.87	60.86	59.65	62.06	9.04	**20.29**	**0.000**
BMI [kg/m^2^]	23.97	23.62	24.32	2.47	21.49	21.12	21.85	2.71	**9.74**	**0.000**
PSQI [scores]	3.38	3.14	3.62	1.72	5.21	4.96	5.47	1.90	**−10.33**	**0.000**
Wholegrain bread [scores]	0.25	0.22	0.29	0.26	0.41	0.37	0.45	0.30	**−5.78**	**0.000**
Milk [scores]	0.43	0.39	0.47	0.29	0.52	0.48	0.56	0.31	**−3.07**	**0.002**
Fermented milk [scores]	0.33	0.30	0.36	0.23	0.52	0.49	0.55	0.23	**−8.17**	**0.000**
Curd cheese [scores]	0.21	0.19	0.24	0.17	0.26	0.23	0.28	0.17	**−2.54**	**0.012**
Fish [scores]	0.10	0.08	0.11	0.09	0.09	0.08	0.10	0.07	0.67	0.506
Legumes [scores]	0.12	0.10	0.15	0.16	0.16	0.14	0.18	0.13	**−2.38**	**0.018**
Fruits [scores]	0.52	0.49	0.56	0.28	0.74	0.69	0.79	0.37	**−6.60**	**0.000**
Vegetables [scores]	0.59	0.55	0.64	0.32	0.88	0.83	0.93	0.38	**−8.24**	**0.000**
Fastfood [scores]	0.18	0.16	0.21	0.15	0.08	0.07	0.09	0.07	**8.88**	**0.000**
Fried meals [scores]	0.54	0.50	0.58	0.31	0.30	0.28	0.32	0.18	**9.93**	**0.000**
Yellow cheese [scores]	0.50	0.47	0.54	0.27	0.34	0.31	0.37	0.23	**6.86**	**0.000**
Sweets [scores]	0.41	0.38	0.44	0.24	0.43	0.39	0.47	0.32	−0.78	0.436
Canned meals [scores]	0.20	0.17	0.24	0.25	0.02	0.02	0.03	0.05	**10.63**	**0.000**
Sweetened beverages [scores]	0.36	0.31	0.40	0.30	0.09	0.07	0.11	0.15	**11.85**	**0.000**
Energy drinks [scores]	0.18	0.15	0.21	0.18	0.10	0.07	0.12	0.17	**4.90**	**0.000**
Alcoholic drinks [scores]	0.13	0.11	0.15	0.15	0.06	0.05	0.07	0.05	**6.32**	**0.000**
IPAQ [MET/minutes/week]	3608.18	3418.49	3797.86	1356.88	3019.81	2886.51	3153.11	1000.89	**5.08**	**0.000**

Footnote: Statistically significant differences are in bold font. BMI—body mass index, PSQI—Pittsburgh Sleep Quality Index, QEB—Questionnaire of Eating Behaviours, IPAQ—International Physical Activity Questionnaire.

**Table 2:** Variable importance statistics obtained from the Boruta algorithm distinguishing good and poor sleep quality. Nine variables were confirmed as significant, primarily representing high-calorie or processed foods and beverages, while seven were rejected. No tentative features remained, confirming the stability of the model.

Variable	Mean Importance	Median Importance	Min Importance	Max Importance	Decision
fastfood	4.22	4.15	2.98	5.67	Confirmed
fried, meals	3.89	3.56	2.1	5.01	Confirmed
sweetened, beverages	3.87	3.54	2.45	4.98	Confirmed
energy, drinks	3.1	3.02	1.88	4.09	Confirmed
alcoholic, drinks	2.94	2.84	1.76	3.98	Confirmed
canned, meals	2.83	2.65	1.95	4.6	Confirmed
vegetables	2.41	2.32	1.5	3.05	Confirmed
curd, cheese	2.11	2.07	1.44	2.88	Confirmed
wholegrain, bread	1.87	1.83	1.21	2.36	Confirmed
milk	0.54	0.5	0.31	0.71	Rejected
fermented, milk	0.46	0.44	0.25	0.66	Rejected
fish	0.39	0.37	0.22	0.58	Rejected
legumes	0.33	0.31	0.18	0.54	Rejected
fruits	0.31	0.3	0.16	0.47	Rejected
yellow, cheese	0.29	0.27	0.15	0.44	Rejected
sweets	0.21	0.2	0.12	0.34	Rejected

Footnote: Variables confirmed by the Boruta algorithm were considered significantly associated with sleep quality category (good vs. poor). Values correspond to the distribution of feature importance scores across 500 random forest iterations. No tentative variables remained after the rough-fix step, indicating a stable model.

**Table 3:** Dietary behaviour variables retained in the LASSO logistic regression model (λ_1_se criterion). Positive β coefficients indicate higher odds of poor sleep quality (risk factors), whereas negative coefficients indicate lower odds (protective behaviours).

Variable	β (Log-Odds)	OR	Direction
sweetened.beverages	0.91	2.49	↑ poor sleep
energy.drinks	0.68	1.97	↑ poor sleep
fastfood	0.58	1.79	↑ poor sleep
fried.meals	0.49	1.63	↑ poor sleep
vegetables	−0.42	0.66	↓ poor sleep
curd.cheese	−0.29	0.75	↓ poor sleep

Footnote: Upward arrows (↑) indicate variables associated with increased odds of poor sleep quality (risk-enhancing factors), whereas downward arrows (↓) indicate variables associated with decreased odds of poor sleep quality (protective factors).

**Table 4:** Moderated mediation of the association between dietary behaviour (MCA) and sleep quality (PSQI) via physical activity (IPAQ), moderated by sex and adjusted for BMI.

Path/Predictor	β	SE	t	*p*
Model a: IPAQ ~ MCA + BMI + Sex				
Intercept	0.35	0.07	5.02	<0.001
MCA (X)	−0.16	0.05	−3.27	0.001
BMI	−0.22	0.05	−4.14	<0.001
Sex (W)	−0.67	0.10	−6.59	<0.001
Model R^2^ = 0.14				
Model b: PSQI ~ MCA + IPAQ × Sex + BMI				
Intercept	3.02	0.12	25.21	<0.001
MCA (X)	−0.11	0.08	−1.35	0.177
IPAQ (M)	−0.20	0.10	−2.05	0.041
Sex (W)	2.42	0.18	13.66	<0.001
BMI	0.91	0.09	10.18	<0.001
IPAQ × Sex (M × W)	−0.45	0.16	−2.78	0.006
Model R^2^ = 0.44				

Footnote: IPAQ—International Physical Activity Questionnaire, MCA—Multidimensional Comparative Analysis, BMI—body mass index, PSQI—Pittsburgh Sleep Quality Index, X—the independent variable, M—mediator, W—moderator (sex). R^2^ represents the proportion of variance explained by the model.

**Table 5:** Conditional indirect effects (bootstrapped, 95% CI).

Group	Indirect Effect (β)	95% CI	*p*-Value
Males	0.032	[0.003, 0.079]	0.021
Females	0.102	[0.023, 0.193]	0.004

Footnote: *p*-values estimated from 5000 bootstrap resamples (two-tailed). Effects are significant when the CI does not include zero.

## Data Availability

The data presented in this study are available on request from the author. The data are not publicly available due to privacy and ethical restrictions related to human participant data.

## Supplementary Materials

The following supporting information can be downloaded at https://www.mdpi.com/article/10.3390/nu18010026/s1, Table S1: Descriptive statistics (mean, standard deviation, median, quartile range and skewness) of the raw data: anthropometric measurements, body mass index, and questionnaires scores: sleep quality (PSQI), sleepiness during day (ESS), dietary behaviours (QEB) and physical activity (IPAQ). Results are presented separately for males and females. Table S2: Firth’s bias-reduced logistic regression for poor sleep quality (PSQI > 5); Table S3: Quantitative indices describing similarity and divergence of dietary clustering structures between good and poor sleepers.
