# Supplementary material for: Sex Moderates the Mediating Effect of Physical Activity in the Relationship Between Dietary Habits and Sleep Quality in University Students
Source: Nutrients. 2025 Dec 20;18(1):26. doi: 10.3390/nu18010026 (PMC12788073; doi:10.3390/nu18010026)
Supplement: Supplementary file 1 [file nutrients-18-00026-s001.zip › Table S1.pdf]

Supplementary Table S1. Descriptive statistics (mean, standard deviation, median, quartile range and skewness) of the raw data: anthropometric measurements, body mass index, and questionnaires scores: sleep quality (PSQI), dietary behavriors (QEB) and physical activity (IPAQ). Results are presented separately for males and females.

Males n = 199	Mean	SD	Me	Q1-Q3	Skewness
Age [y]	20,73	0,85	20,76	0,98	0,57
Body height [cm]	182,19	7,10	81,20	10,50	-0,11
Body weight [kg]	79,63	9,87	79,00	13,50	0,65
BMI [kg/m^2]	23,97	2,48	23,54	3,01	0,74
PSQI [pts]	3,38	1,72	3,00	2,00	0,97
Wholegrain bread [pts]	0,29	0,33	0,16	0,44	2,51
Milk [pts]	0,50	0,38	0,50	0,41	1,57
Fermented milk [pts]	0,37	0,29	0,35	0,36	2,07
Curd cheese [pts]	0,23	0,19	0,18	0,29	1,19
Fish [pts]	0,10	0,11	0,07	0,08	4,26
Legumes [pts]	0,14	0,19	0,06	0,14	5,29
Fruits [pts]	0,61	0,38	0,50	0,33	1,45
Vegetables [pts]	0,71	0,43	0,54	0,50	1,32
Fastfood [pts]	0,20	0,17	0,14	0,20	1,88
Fried meals [pts]	0,64	0,41	0,50	0,44	1,41
Yellow cheese [pts]	0,59	0,36	0,51	0,37	1,06
Sweets [pts]	0,47	0,31	0,50	0,27	1,86
Canned meals [pts]	0,23	0,29	0,13	0,31	1,60
Sweetened beverages [pts]	0,41	0,37	0,38	0,49	1,43
Energy drinks [pts]	0,20	0,21	0,14	0,24	1,66
Alcoholic drinks [pts]	0,14	0,19	0,08	0,11	5,75
IPAQ [MET/minutes/week]	3608,18	1356,88	3364,57	1949,50	0,45
Females n = 219	Mean	SD	Me	Q1-Q3	Skewness
Age [y]	20,56	20,52	0,97	0,74	0,31
Body height [cm]	168,17	167,90	7,30	6,01	0,30
Body weight [kg]	60,86	59,10	11,00	9,05	0,88
BMI [kg/m^2]	21,49	21,11	3,55	2,71	0,81
PSQI [pts]	5,22	5,00	2,00	1,90	0,07
Wholegrain bread [pts]	0,48	0,50	0,53	0,37	0,93
Milk [pts]	0,61	0,59	0,49	0,39	0,73
Fermented milk [pts]	0,60	0,50	0,27	0,30	0,76
Curd cheese [pts]	0,28	0,27	0,31	0,20	0,80
Fish [pts]	0,10	0,06	0,08	0,08	2,93
Legumes [pts]	0,17	0,14	0,18	0,15	1,61
Fruits [pts]	0,91	0,98	0,76	0,52	0,46
Vegetables [pts]	1,11	1,00	1,00	0,56	0,24
Fastfood [pts]	0,09	0,06	0,08	0,08	3,10
Fried meals [pts]	0,33	0,33	0,36	0,22	2,26
Yellow cheese [pts]	0,38	0,43	0,36	0,28	1,96
Sweets [pts]	0,50	0,50	0,35	0,43	2,08
Canned meals [pts]	0,03	0,00	0,05	0,05	6,58
Sweetened beverages [pts]	0,09	0,06	0,13	0,19	5,94
Energy drinks [pts]	0,11	0,06	0,14	0,21	4,75
Alcoholic drinks [pts]	0,06	0,06	0,03	0,06	4,20
IPAQ [MET/minutes/week]	3019,81	2849,00	1036,50	1000,89	0,98

Footnote: SD – standard deviation, BMI – body mass index, PSQI - Pittsburgh Sleep Quality Index, IPAQ – International Physical Activity Questionnaire, QEB – Questionnaire of Eating Behaviours
