# Supplementary material for: Sex Moderates the Mediating Effect of Physical Activity in the Relationship Between Dietary Habits and Sleep Quality in University Students
Source: Nutrients. 2025 Dec 20;18(1):26. doi: 10.3390/nu18010026 (PMC12788073; doi:10.3390/nu18010026)
Supplement: Supplementary file 1 [file nutrients-18-00026-s001.zip › Table S2.pdf]

Supplementary Table S2. Firth's bias-reduced logistic regression for poor sleep quality (PSQI > 5).

**Dietary behaviour**	**OR**	**β = ln(OR)**	**95% CI**	**p-value**	**Direction**
sweetened beverages	0.51	-0.67	0.15–1.56	0.245	↓ poor sleep
energy drinks	1.90	0.64	0.51–6.78	0.326	↑ poor sleep
fast food	3.07	1.12	0.36–24.65	0.301	↑ poor sleep
fried meals	1.80	0.59	0.66–4.83	0.248	↑ poor sleep
vegetables	1.11	0.10	0.57–2.18	0.754	= no associations
curd cheese	0.22	-0.65	0.05–0.86	0.030	↓ poor sleep

Footnote: Predictors correspond to dietary behaviours retained by the LASSO model (λ_{1se}). Firth's bias-reduced logistic regression was used as a sensitivity analysis; estimates are conservative and confidence intervals are wide due to limited event counts.
