# Supplementary material for: Sex Moderates the Mediating Effect of Physical Activity in the Relationship Between Dietary Habits and Sleep Quality in University Students
Source: Nutrients. 2025 Dec 20;18(1):26. doi: 10.3390/nu18010026 (PMC12788073; doi:10.3390/nu18010026)
Supplement: Supplementary file 1 [file nutrients-18-00026-s001.zip › Table S3.pdf]

Supplementary Table S3. Quantitative indices describing similarity and divergence of dietary clustering structures between good and poor sleepers.

Index	Description	Value	Interpretation
Cophenetic correlation (good sleepers)	Internal coherence of dendrogram	0.42	Moderate clustering consistency
Cophenetic correlation (poor sleepers)	Internal coherence of dendrogram	0.23	Lower clustering coherence
Baker's Gamma	Rank similarity between dendrograms	0.31	Partial similarity
Entanglement coefficient	Degree of crossing in tanglegram	0.73	Moderate–high divergence
Mantel test (r)	Global similarity of distance matrices	0.90	High overall similarity
Mantel test (p)	Statistical significance	<0.001	Significant

Footnote: Cophenetic correlations reflect internal consistency of hierarchical clustering within each group. Baker's Gamma and the entanglement coefficient quantify similarity and divergence between clustering structures, while the Mantel test assesses global correspondence between distance matrices. These indices were used for descriptive purposes to support the qualitative interpretation of dietary pattern organisation
